# Diagnostic Markers of User Experience, Play, and Learning for Digital Serious Games: A Conceptual Framework Study

**DOI:** 10.2196/14620

**Published:** 2019-07-16

**Authors:** Jun Wen Tan, Nabil Zary

**Affiliations:** 1 Games for Health Innovations Centre Lee Kong Chian School of Medicine Nanyang Technological University Singapore Singapore; 2 Mohammed Bin Rashid University of Medicine and Health Sciences Dubai United Arab Emirates

**Keywords:** serious games, diagnostic criteria, medical education

## Abstract

**Background:**

Serious games for medical education have seen a resurgence in recent years, partly due to the growth of the video game industry and the ability of such games to support learning achievements. However, there is little consensus on what the serious and game components in a serious game are composed of. As a result, electronic learning (e-learning) and medical simulation modules are sometimes mislabeled as serious games. We hypothesize that one of the main reasons is the difficulty for a medical educator to systematically and accurately evaluate key aspects of serious games.

**Objective:**

This study aimed to identify markers that can evaluate serious games and distinguish between serious games, entertainment games, and e-learning.

**Methods:**

Jabareen’s eight-phase framework-building procedure was used to identify the core markers of a serious game. The procedure was modified slightly to elicit “diagnostic criteria” as opposed to its original purpose of a conceptual framework. Following the identification of purported markers, the newly developed markers were tested on a series of freely available health care serious games—Dr. Game Surgeon Trouble, Staying Alive, and Touch Surgery—and the results were compared to the published test validity for each game.

**Results:**

Diagnostic criteria for serious games were created, comprising the clusters of User Experience (UX), Play, and Learning. Each cluster was formed from six base markers, a minimum of four of which were required for a cluster to be considered present. These criteria were tested on the three games, and Dr. Game Surgeon Trouble and Staying Alive fit the criteria to be considered a serious game. Touch Surgery did not meet the criteria, but fit the definition of an e-learning module.

**Conclusions:**

The diagnostic criteria appear to accurately distinguish between serious games and mediums commonly misidentified as serious games, such as e-learning modules. However, the diagnostic criteria do not determine if a serious game will be efficacious; they only determine if it is a serious game. Future research should include a much larger sample of games designed specifically for health care purposes.

## Introduction

The past decade has seen a surge of digital games seeking to educate, train, or otherwise inform their players in a broad spectrum of topics [1]. These serious games are often defined as games developed for nonrecreational purposes and are frequently deployed as adjuncts to education and therapy [2,3]. Documented uses of serious games include teaching mathematics to young children, motivating the elderly to exercise, and increasing surgical competencies of medical students and residents [4-7]. Although such games are unable to replace full-time educators, they are effective at reinforcing concepts learned through conventional teaching methods [8].

This reinforcement is particularly notable in fields characterized by intensive, high-volume learning, such as medical education. Perhaps, due to the ubiquity of game playing in the present day, where video games generate astronomical levels of revenue, it is no surprise that educators are seeking to bend the vast potential of gaming toward serious causes like learning [3,9,10]. This shift in paradigm comes at the heels of the once ironclad assumptions linking the notions of play with time-wasting and frivolousness [1,11]. As more serious games begin featuring in institutions across the globe, they receive ever-increasing attention from stakeholders seeking to leverage the benefits of serious games—be it for profit or learning.

However, despite the growing interest in serious games and the vast array of such games being pushed to market, there remains little consensus on core components that afford a serious game its seriousness while also retaining the fun and entertaining elements that characterize recreational games [3,12,13]. Many serious games are affected by a variety of issues that impede the said serious agenda. Although such games are often the result of attempts to gamify an existing teaching method, these often suffer from poor instructional or game design and thus perform poorly as compared to methods they were meant to replace or support [14,15]. Surprisingly, the reverse is also true: When educational elements are inserted into near-completed games as an afterthought, they do not necessarily perform well [16].

For an educational serious game to be effective, the educational content must be robust, appropriate for the target audience, and well-integrated into the game [17,18]. Thus, it comes as no surprise that many serious games are now subject to rigorous validation studies long before implementation.

Recent reviews of the literature suggested that the vast majority of research remains dedicated to testing the efficacies of specific serious games for their intended purposes and, where applicable, the cognitive processes activated by playing these games [3,4,6,19,20]. Often, newly developed games are trailed to a targeted sample, typically with a control group, and game efficacy is determined by whether the game achieved its intended result, such as increases in standardized test scores. This user-centric focus, while meritorious, often does not addressed the factors, mechanisms, or processes that afford serious games efficacy and receptivity by their target audiences. It leaves the following question unanswered: “How do I know this serious game will be both serious and a game?”

Consequently, this has resulted in a gap in the literature with regard to consistent, definitive, and validated diagnostic criteria to serve both as a reference and basis of evaluation for serious games. This has impeded the development of new serious games whose topics have not yet been subject to extensive testing and the objective-appropriate selection of ready-made games already available on the market. The lack of criteria is especially punishing for educational pathways featuring intensive study, such as medical institutions, that may be seeking additional educational strategies to enhance student engagement and quality of learning.

The development and acquisition of serious games are costly, time-consuming endeavors, especially in the context of health care. Often, the initiators of serious game development are health care professionals idealizing games to tackle specific health or educational challenges, while the actual game makers are technical specialists with comprehensive knowledge of game development. The absence of practical guidelines, or diagnostic criteria, risks the production of ineffective serious games which, in turn, is compounded by the poor allocation and utilization of resources and leads to institutions being discouraged from adopting games that might significantly enhance the performance of their students.

Although Yusoff et al [21] and Rooney [22] have proposed two frameworks established for use by game designers and educators, both were designed for use during the game development process, as opposed to the validation of ready-made games.

The framework by Yusoff et al [21] combined learning theory with gaming requirements to ensure games meet learning outcomes [21]. The Learning Activity, built from the intended learning outcomes and game aspects that support learning and engagement, was key to this framework. It acted with the game’s genre and could be modified based on feedback derived from a player’s achievement within the game. However, the framework acts as a guiding tool during the developmental phase of game design and does not readily function as a validation framework. The theoretical bases of the framework also require that users possess familiarity with either or both game development and pedagogy and may not be used easily by prospective game producers unfamiliar with either.

Rooney [22] proposed a triadic interaction of play, pedagogy, and fidelity that together form a framework for serious game design in higher education. They discussed the theoretical underpinnings and key literature and challenges addressed. Although play and pedagogy referred to game play and the pedagogical aspects of learning, fidelity was defined as the extent to which the game emulated the real world, both physically, such as visual displays and behaviors of physics engines, and functionally, as the extent to which the game behaves like the real world in response to player actions. However, Rooney [22] acknowledged difficulties in balancing the framework’s components, in part, due to the multidisciplinary and sometimes competing nature of game design that has thus far prevented reconciliation of the components into a coherent theoretical framework. In addition, no validation of the framework, such as designing a game from the ground up, was provided.

Although several frameworks related to serious games have been proposed, all require above-average competencies in the knowledge of game development and are applicable in stages of game development. While touched upon, both frameworks did not define the base markers comprising serious games, were not validated against the existing ready-made serious games, and were not created with specific relevance to or confirmed to be compatible with medical and medical education games. In this context, none are easily distilled into the base components of a serious game.

Therefore, this study aims to deconstruct serious games into their base markers before validating them and an accompanying diagnostic criteria for evaluating serious games that can be used by nongaming experts. The proposed “diagnostic criteria” would enable the validation of ready-made serious games and act as a guide to ensure newly created serious games are both serious and games. The study’s objectives are hence twofold. The study will first detail the procedure used for the deconstruction of serious games into their base markers. These markers will then be tested on three established serious games to ensure that the diagnostic criteria are able to assess the serious and game aspects of serious games.

## Methods

### Deconstruction of Serious Games Into Base Markers

**Figure 1:**
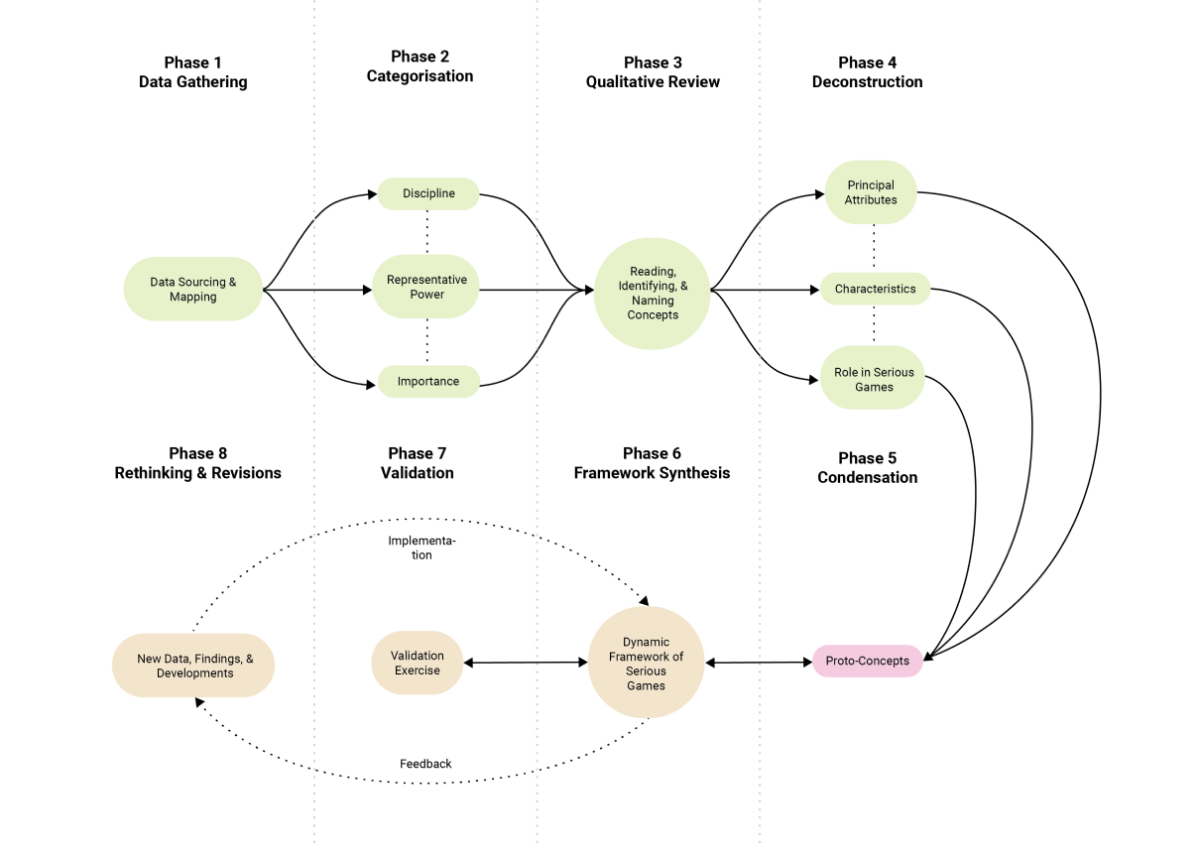
Overview of Jabareen’s eight phases [23].

Figure 1 shows the eight-phase procedure by Jabareen [23] for building a conceptual framework that was used for the isolation of base markers.

In phase 1, sources of serious game data were extensively mapped. Sources consisted of multidisciplinary journal articles reporting on evaluative research on serious games, consultations with educators experienced in game-based teaching, and auditing of learning-through-play workshops during international conferences at the Lee Kong Chian School of Medicine in Singapore. In phase 2, the accumulated data were extensively reviewed and categorized according to discipline, representative power, and importance. In phase 3, a qualitative inquiry into the data was conducted. Concepts that surfaced were identified and named even if they competed with or contradicted each other. In phase 4, concepts were deconstructed to identify their main attributes, characteristics, and roles in serious games. Deconstructed concepts were then sorted by their ontological, epistemological, and methodological roles. In phase 5, concepts with similar features were condensed, reducing the total number of concepts to more manageable levels. In phase 6, using an iterative process, the newly integrated concepts were synthesized into a single theoretical framework. Concepts were subject to repeated resynthesis until a sensible general theoretical framework was recognized. In the study, three clusters of interrelated serious games markers were produced instead of a framework. In phase 7, we examined three established serious games to determine if the markers proposed by Jabareen’s process can be confirmed in games via the detection of each cluster’s base markers. Finally, in phase 8, as theoretical frameworks representing multidisciplinary phenomena remain dynamic, the tested framework will eventually be revised in light of new findings and developments as part of future studies undertaken by the authors and the broader serious games scientific community.

### Games Used to Validate the Framework

Three serious games for medical education were used in the validation of the clusters and their base markers. All three games had been previously validated as serious games efficacious in their target areas or audiences and are easily accessible by medical students and professionals, as they are freely available from either the Android or iOS mobile app stores or from the developer’s website [24-26].

“Touch Surgery” simulates hundreds of different medical procedures in three-dimensional environments and enables users to train in a three-dimensional environment. Upon selection of a procedure, users are guided through all the appropriate steps of an operation step-by-step, following which, players are provided opportunities for rehearsal and self-assessment via multiple-choice questions that focus on cognitive decision making. Of the available modules, the chest tube insertion procedure was chosen due to its relative novelty to the investigators of the study.

“Dr Game, Surgeon Trouble” trains medical residents to recognize and correct responses to equipment failure events during laparoscopic surgery. Player attention is held by a match-three-puzzle minigame unrelated to surgery and must concurrently solve equipment-related problems in a visually embedded laparoscopic tower. The onset of problems is accompanied by signals such as camera blurring or changes in lighting intensity, which occur partially beyond a player’s direct focus of attention, emulating a surgical environment. Upon detection of a problem, the player is then moved to a troubleshooting mode where the educational aspect of the game features. At this stage, players interact with the laparoscopic tower to resolve equipment malfunctions and are given a set number of “attempts” at the correct solution. Throughout, the game maintains a cycle of challenges, actions, and direct feedback.

“Staying Alive” teaches the general public and health professionals about the management of a sudden cardiac arrest. Players are presented a three-dimensional environment wherein a man has just collapsed due to the onset of cardiac arrest and is guided through the appropriate measures and techniques required to maximize his chances of survival. Difficulty increases across levels, where level 1 takes place in an indoor office, while level 2 takes place in a sports field.

### Validation of the Markers

In the validation phase of the study, each game was played till completion and markers that surfaced were noted using the Serious Games Markers Scoring Protocol (Multimedia Appendices 1 and 2). As the markers serve as a means to detect the base markers of the game, as opposed to how well or how much of a marker is represented, a binary scoring system was utilized. Markers that clearly featured in games were marked as present and their total scores were tallied.

## Results

### Markers of Serious Games

**Figure 2:**
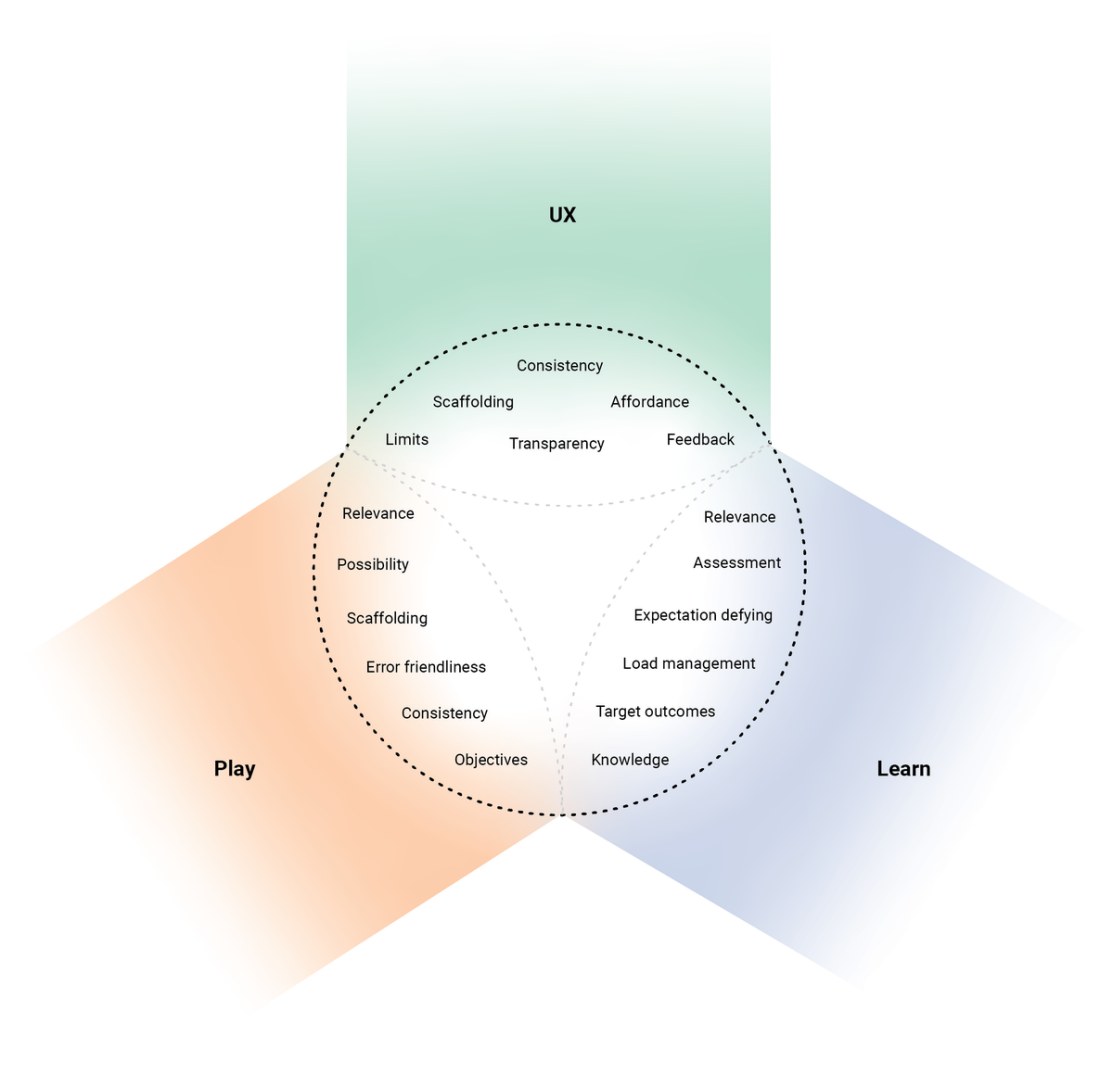
Overview of the diagnostic criteria of serious games. UX: User Experience.

Figure 2 shows the three clusters of markers that, when brought together, form the base composition of a serious game—User Experience (UX), Play, and Learning—that must be present for a serious game to be considered both serious and a game. This is in contrast to recreational games and e-learning, which are respectively missing the clusters of Learning and Play.

UX encompassed the player’s cognitive and affective experiences while playing and interacting with the game and found ways in which they were moderated by the game’s actual play, professional applicability, and usability through its user interface. The cluster comprised elements detailed in Textbox 1.

The cluster of Play encompassed the game’s nonlearning mechanics, genres, style, control mechanism (ie, controller and keyboard), within-game objectives, playable content, and infrastructure (ie, cloud-based, hardware requirements). The cluster comprised markers detailed in Textbox 2.

Learning encompassed the scenes, scenarios, or situations wherein the player is exposed to the knowledge or skills the game intends to impart. They need not be singular, and distinct instances can be spread out or embedded into the game’s central mechanics. The cluster comprised markers detailed in Textbox 3.

**Markers comprising the cluster of User Experience.**

- Limits: The game attempts to govern how users should play, in accordance with the developer’s intent, and discourages styles that circumvent its overall purpose. It also refers to constraining the player within the play area to avoid unintended bugs.
- Feedback: For most of the time, the game responds to actions taken by the player, confirming to the player that the said action had taken place. It typically refers to auditory or visual cues in response to all actions, no matter how inconsequential, such as clicks or bumps when interacting with objects.
- Scaffolding: The game gradually increases the intensity of mechanics that directly influence player impressions and feelings toward it in the moment of play. It also refers to the gradual introduction of new mechanics across time, if such mechanics are available.
- Affordance: Actions or purposes available to the player should be affordable, in that the game makes clear how the presented options are to be used, or such that the player will be able to figure out how options are to be used. Does not apply to puzzle-based games or objectives that require significant cognitive input.
- Transparency: Options available to the player, in both gameplay and the game’s user interface, require little to no thought to discern their purpose. Does not apply to mechanics designed to assess levels of competencies and novel mechanics accompanied by dedicated explanations.
- Consistency: Game manages its interactions with the player in a mostly consistent manner, inclusive of predictable outcomes when using external instruments (mice, keyboards, etc) to interact with the game.

**Markers comprising the cluster of Play.**

- Relevance: The game’s overall themes, style, genre, and design should be professionally applicable to its target audience at the time of publication.
- Objectives: The game possessed explicit overarching or instance-specific purposes for the player to attain.
- Possibility: The game should introduce more substance, usually new game content, events, and features over time. These may include new methods to overcome challenges, and entirely novel materials or themes within the game.
- Consistency: Methods, controls, game mechanics, and rules for gameplay should display consistency most of the time. Does not consider the actual playable content of the game.
- Scaffolding: The effort or cognitive input required by a player to overcome challenges or content presented by the game should gradually increase as play time increases. Play should begin at a manageable level before increasing in difficulty to maintain challenge and flow. If difficulty is moderated by variations of in-game mechanics, then these mechanics should be gradually introduced.
- Error Friendliness: The game allows for player errors to be made, allows consequences of said errors, and does not bar an action or cease missions upon detection of an incorrect choice.

**Markers comprising the cluster of Learning.**

- Target Outcomes: The game has clear goals or target outcomes it intends to achieve. It can be operationalized as intent to raise latent or constant awareness of a problem. If targeted at behaviors, it can be operationalized as behavioral modification attempts.
- Knowledge: The game contains the knowledge and skills it intends for the player to take in and utilize.
- Relevance: Knowledge or skills present in the game have been set to a standard suitable for the target audience’s learning level and interests, wherein interest is defined as whether the content will be applicable to the said audience.
- Cognitive Load Management: The game supports the player’s exposure to new knowledge and allows for the regular intake of new information. The game ideally seeks to maintain players within the zone of proximal development, avoids overloading the player, and ensures that the pace is not too slow as to induce boredom or disinterest.
- Expectation Defying: The game attempts to prevent players from being conditioned to one stimulus to the point that introducing a second, necessary stimulus has no effect. Operationally, the game avoids monotonous and predictable “learning moments” and takes steps to keep players from knowing what will happen next with regard to learning.
- Assessment: The game features a system to assess player learning improvements with regard to the target learning outcomes. It need not be operationalized as traditional scorecards and may feature as achievement or medal systems. “Total Score” features that lack clarity and specifics do not qualify.

### Scoring Methodology

Due to the need for a simple validation process, each marker of each cluster is marked on a binary level—they are either present (1) or not present (0)—and must be indisputably present in a game to be considered so. Each cluster (UX, Play, and Learning) contains six markers and will be scored from 0 to 6 depending on the number of markers present, with a score of ≥4 denoting a cluster as present. When all clusters score ≥4, the game is considered a serious game.

The serious game to be validated is to be played from start to finish or, for games designed to end midway due to player error, until five game-ending mistakes are committed or until the player is no longer willing to continue due to the inability to overcome the obstacle wherein the mistakes were committed, whichever comes first. The player is to observe the game for each cluster’s markers and record them accordingly.

To facilitate the ease of reporting game scores posttabulation, scores are reported in a UX/Play/Learning format, abbreviated as #U/#P/#L, where # ranges from 0 to 6 depending on the number of markers recorded for the respective clusters.

### Scoring the Seriousness of the Games

#### Game Scores

“Touch Surgery” scored 6U/0P/5L. It was found to possess all six markers of UX and five markers for Learning, missing out on Expectation Defying due to the absence of conditioning stimuli in the Learning cluster of the game. However, the game scored 2 points in the cluster of Play, as the “game” component resembled e-learning as opposed to games defined by Alvarez and Djaouti [2].

“Dr. Game Surgeon Trouble” scored 6U/5P/6L. It was found to possess all six markers of UX and Learning, but missed out the Scaffolding component in Play due to the study being unable to confirm if its match-three-puzzle minigame increased in difficulty over time nor did the frequency of problem signaling increasing over time.

“Staying Alive” scored 6U/5P/5L. It was also found to possess all six markers of UX but missed out on the Possibility component in Play because the second level was mostly the same as the first and there was uncertainty about whether there was an increase in difficulty.

**Figure 3:**
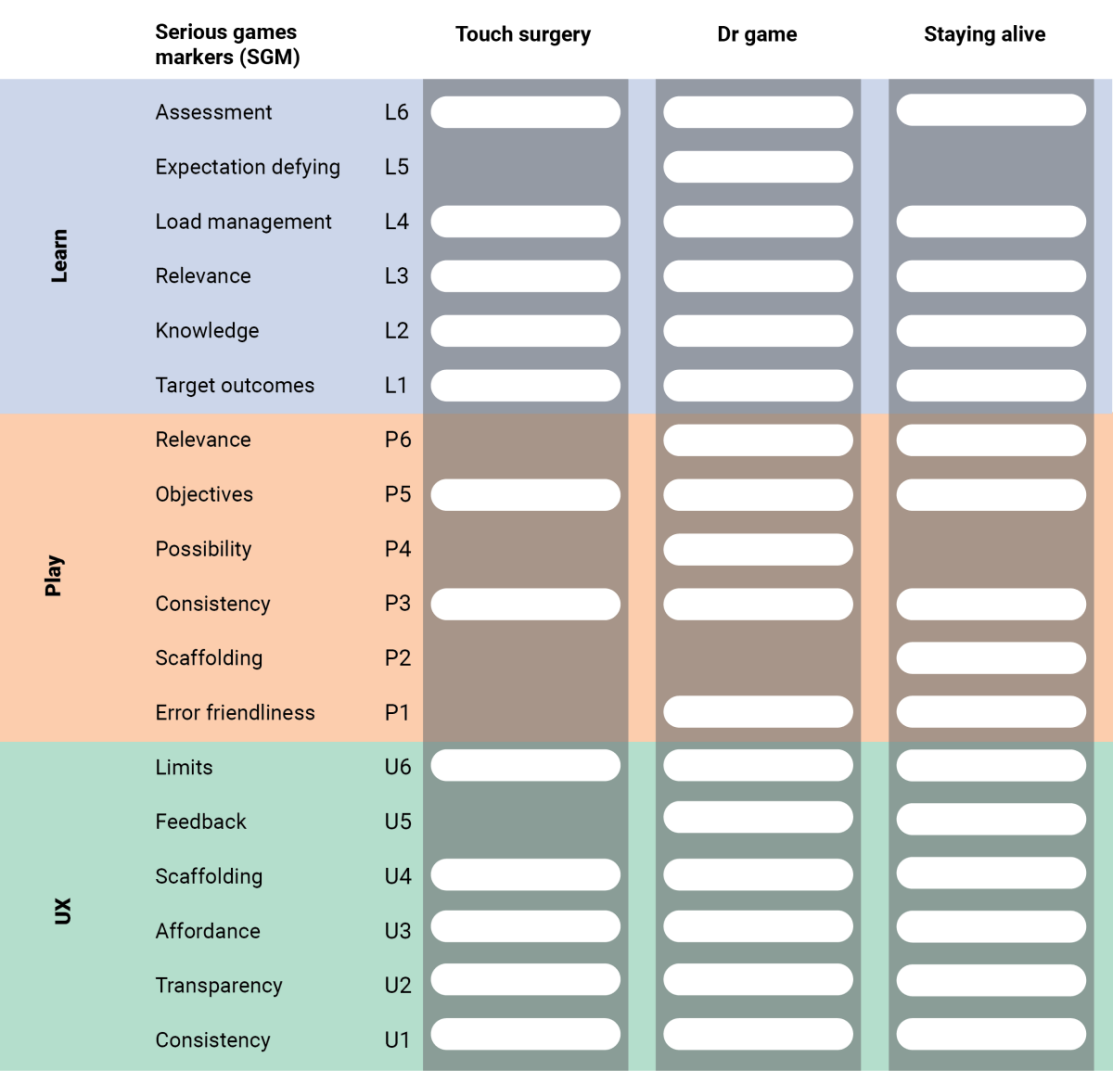
Markers for the three games evaluated. UX: User Experience.

Figure 3 summarizes the game scores for the three games.

## Discussion

### Principal Findings

The diagnostic markers of serious games were developed by an iterative process that began with the identification of knowledge within the existing literature. During this process, the two existing frameworks of serious games proposed by Yusoff et al [21] and Rooney [22] were found to be used during the game development process and not as a validation tool for ready-made games [21,22]. As no such tool existed, this study attempted to close the gap in the literature through the construction and validation of diagnostic markers that included extensive deconstruction and resynthetization of data until a sensible cluster of markers was recognized. The newly developed clusters were then tested on a series of ready-made health care serious games. Although all games were previously validated by at least one study, some appeared to defy the criteria’s definition of a serious game.

Haubruck et al [25] conducted a randomized controlled trial that demonstrated the validity and effectivity of “Touch Surgery” as an educational adjunct for chest tube insertion procedures [25]. The study applied the Gameplay/Purpose/Scope (G/P/S) classification model by Djaouti et al [13] to confirm Touch Surgery’s status as a serious game. However, the G/P/S model aims to precisely classify a serious game into a subgenre via a system that acknowledges both the “serious” and “game” dimensions. The model’s principal goal was to allow educators to find serious games that were useful for their specific causes but were otherwise not designed for or marketed toward their use. A secondary application of the model was to identify entertainment games that could be repurposed for use in education. In both circumstances, the model requires that the game being classified qualifies as one to begin with.

Similarly, notwithstanding the study by Haubruck et al, no citation referring to Touch Surgery as a serious game could be found, including the manufacturer’s website. This discrepancy is most likely due to a case of mistaken identity due to the multitude of definitions for what exactly construes a serious game. Djaouti et al [13] noted that serious gaming draws expertise from a broad range of fields such as pedagogy, computer science, and medicine that may not wholly agree with the definition of a serious game [13].

Alvarez and Djaouti defined “serious games” for health care as games that were designed specifically for the serious purpose of providing education via digital devices [2]. The term “serious gaming” was defined as the use of any digital games for health care education and could also refer to nonserious games used for a serious purpose, which they termed “serious diverting” [2]. Therefore, although none of the definitions define what construes a game, it can be inferred that the related software must be games to begin with. A related concept (“gamification”) was defined as the use of game elements in real-world applications that typically were not games to begin with [27]. Such characteristics include rewarding users of e-learning with points, badges, or achievements upon completion of a module. As “Touch Surgery” does not demonstrate transparent implementation of game elements, does not offer users challenging problems outside of its assessment module, and was not cited as a serious game by its manufacturer, it is more applicably categorized as an e-learning tool than a serious game, thereby falling in line with the markers and proposed diagnostic criteria, notwithstanding its efficacy as an educational tool.

Conversely, “Dr. Game Surgeon Trouble” appears to fit well with the proposed criteria for a serious game, and its status as one is supported by existing literature. Graafland et al [24] had developed the game specifically to improve the problem recognition and resolution skills of surgical trainees [24,28]. The game has had its construct validity established, and a randomized controlled trial demonstrated favorable outcomes in improving trainee problem recognition and resolution. Moreover, while there is no evidence to suggest that Graafland et al [24] employed existing conceptual frameworks of serious games during the development phase, the game demonstrates the distinct, yet well-integrated game and educational components that fit neatly within common definitions of serious games. Although requiring only a few minutes to complete, “Staying Alive” appeared to fit well within the diagnostic criteria, and its status as a serious game is supported by both the manufacturer and a randomized controlled study by Drummond et al [26].

Validating all three games through either Yusoff’s [21] or Rooney’s [22] conceptual frameworks would logically require that both first be adapted for use in ready-made games. Such a venture, while plausible, is likely a complex undertaking that requires some degree of familiarity with game development, game playing, and related pedagogies such as learning through play or game-based learning in addition to validating the suitability of the adapted frameworks or diagnostic criteria. Instead, due to the differing goals of each, a more appropriate use would be their original purposes: Yusoff’s [21] framework to ensure newly developed serious games can meet their learning outcomes and Rooney’s [22] framework to ensure a balance of design and pedagogical elements during game development.

### Limitations

The diagnostic markers for serious games were designed with an aim of simplicity and this, in turn, also serves as one of its limitations. In this regard, the markers may only be used to discriminate between serious games and other digital solutions purporting to be serious games, but belonging to other fields like e-learning. These markers, and the associated diagnostic criteria, cannot determine the efficacy of a serious game, as demonstrated by the analysis of “Staying Alive,” a serious game that did not perform as well as its creators had hoped as compared to a conventional e-learning alternative.

Another limitation is the lack of readily available, high-quality health care serious games to test the markers. Owing to the specific or controlled uses of health care serious games, many remain noncommercialized or unmaintained and thus difficult to acquire. The study was also unable to utilize serious games that required specialized or customized hardware due to logistical constraints.

### Conclusions

The diagnostic markers of serious games presented in this study offer a simpler alternative that may be used by professionals or educators without extensive familiarity with serious games. They allow for the validation of ready-made games on the market and are used to confirm the presence of “seriousness” in any given health care serious game.

Although the markers may also be used during the game development phase to ensure the end product is both serious and a game, further evaluation is required to confirm its validity in this regard. The dynamic nature of diagnostic markers and the accompanying criteria, akin to the those employed by modern medicine, could potentially see new, possibly industry-specific markers being developed from evidence surfacing in future works, which might result in criteria that enable educators or professionals to determine the efficacy of premade serious games while maintaining their simplistic approach to game validation.

## Appendix

Multimedia Appendix 1 Serious Games Markers Scoring Protocol (analog).

Multimedia Appendix 2 Serious Games Markers Scoring Protocol (digital).

## Abbreviations

UX: User Experience
