# Supplementary material for: Diagnostic Markers of User Experience, Play, and Learning for Digital Serious Games: A Conceptual Framework Study
Source: JMIR Serious Games. 2019 Jul 16;7(3):e14620. doi: 10.2196/14620 (PMC6664662; doi:10.2196/14620)
Supplement: Multimedia Appendix 1 [file games_v7i3e14620_app1.docx]

## Appendix I

**SGMSP Scoring Instructions:**

Sum the scores from the 6 items within each of the 3 aspects. If an aspect gains a score of 4 or higher, mark that aspect as 1 (present). If an aspect scores 3 or lower, mark that aspect as 0 (not present). A game must score a total of 3 aspect points to be considered a serious game.

**SERIOUS GAME MARKERS SCORING PROTOCOL**

**PLAY**

Refers to the game’s actual gameplay. Includes the game’s mechanics, genre (e.g. platformer, role-playing, text-adventure), style, controls (e.g. keyboard, mouse, controller), objectives, and infrastructure.

**P1. RELEVANCE:**

1. On a whole, the game’s overall themes, style, genre, and design should logically be applicable to, or be known to applicable to, its target audience at the time of intended publication.
   - Game designed for medical students, which include complex medical terminologies with minimal explanations, should not be released to primary school children where ‘big words’ may impede understanding of gameplay.
   - Geriatric patients who have never used computers before should not be introduced to complex games that require pre-existing knowledge of mouse, keyboard, and UI use (e.g. Crysis 3), and instead be introduced to something simpler like Solitaire (which had the original intent on users to use a mouse).

**P2. OBJECTIVES:**

1. Game possesses a clear overall purpose that can be either hard or soft, overarching, or instance-specific. Hard objectives refer to explicit challenges pertaining to events within the game itself. Soft objectives refer to generalisable tasks. Sandbox games that allow for the creation and completion of player-determined objectives are counted. Discount from assessment objective clearly linked to learning or educational assessments.
   - In Mario, the hard objective is to complete all levels and save Princess Peach.
   - In Cities Skylines, the hard objective is to build a bustling, preferably happy, city with a set of starting funds. The soft objective is to build the city of your liking with available resources, however that may be defined.

**P3. POSSIBILITY:**

1. For most of the time, game levels and available content changes as play time increases. New content, events, and features should become available. In-game events, content, and methods to overcome challenges or obstacles, such as battles, should differ.
   - In Don’t Starve (and Don’t Starve Together), higher level items, enemies, environments are entirely different from their lower level counterparts (not ‘upgrades’ of existing items ala Minecraft)
   - Character, weapon, and vehicle variety increases the more levels completed for the Metal Slug series (e.g. progressing from commandeering tanks early game to flying aircraft by late game).

**P4. CONSISTENCY:**

1. The methods, controls, base level design, game mechanics, and rules for playing the game display consistency most of the time. Discount from assessment the actual content of the game.
   - The original Mario was played and controlled much the same way from start to finish. Jump on, or throw fireballs at most enemies to defeat them, and navigate through the game world through the up-down-left-right controls.
   - Does not apply to consistency in game content (e.g. challenges unfolding in exactly the same way). A game’s theme can be about fruits, but the player should not be presented solely with apples and pears.

**P5. SCAFFOLDING:**

1. For most of the time, the effort or cognitive input required by the player to overcome challenges or content presented in the game should gradually increase as play time increases such that play begins at a manageable level before increasing to maintain challenge and flow. If difficulty is moderated by variations in game mechanics, then mechanics should be progressively disclosed. Discount from assessment how the player is expected to feel as play time increases.
   - Most games being this way – challenges are simple and easier to overcome at the beginning, and gradually increase in difficulty as time progresses. Flow maintenance is often supplemented by the scaling up of player resources (i.e. equipment, abilities) to ensure opposing challenges can still be overcome.
   - Dynasty Warriors Series – enemies do not hit harder across time, but have more health and take longer to kill. Player health and damage does scale, but ultimately player survivability increases much more rapidly than killing potential, breaking flow.

**P6. ERROR FRIENDLINESS:**

1. Game allows for player errors to be made, for there to be consequences of said errors, and does not bar an action or immediately cease the game/mission upon detection of an ‘incorrect choice’. If the game ends after a set number of ‘lives’ are spent, this qualifies for error friendliness. Discount from assessment errors made as part of an educational objective unless said objective is interwoven with gameplay objectives or player choices.
   - In Morrowind, accidentally failing a pickpocket or failing to protect the person you’re escorting does not end the game, but opens up alternative methods of completing the overarching mission/quest.
   - Examples of games with no error friendliness – Crysis 2. If you fail to complete an objective in the given timeframe, the objective fails and you have to restart from a save point.

**SERIOUS GAME MARKERS SCORING PROTOCOL**

**UX**

Refers the elements that interact with the player and moderate their thoughts, emotions, and attitudes toward the game while they are playing it. Includes interactions with the game in terms of its gameplay, professional applicability, usability, usually through its user interface.

**U1. LIMITS:**

1. The game makes attempts to govern how users should play the game according to the developer’s intent, or discourages styles and experiences that circumvent the overall purpose of the game, without suppressing the player to the extent they are disallowed from even slight deviation from the intended experience.
   - Enemies in Metal Gear Solid V react to your methods over time and will counter them after some time (wearing helmets if you keep shooting them in the head, laying traps if you keep sneaking into bases) to discourage you from refusing to experiment with new strategies.
   - XCOM 2. Players of the first XCOM always played cautiously as it was the most efficient way to play. To ‘force’ excitement, XCOM 2 introduced turn limits to force players to make risky moves and suffer the consequences that occur to their soldiers – as was the developer’s original intent.

**U2. FEEDBACK:**

1. Most of the time, the game provides a cue that signals that actions, no matter how inconsequential, taken by the player have occurred. Typically these cue have no effect on gameplay and are often operationalised as sound effects when jumping, interacting with an object, and scrolling through menu options.
   - In Pokemon, when you walk into a wall, there is a ‘bump’ sound effect. In Mario, when you jump there is a ‘sproing’ sound effect.
   - Most shooter games. When you fire a weapon, there will be some kind of bang sound accompanied by a flashing effect on screen.

**U3. SCAFFOLDING:**

1. Game steadily increases the intensity of mechanics that directly influence player impressions and feelings toward it in the moment of play. Also refers to the gradual introduction of new mechanics across time, if such mechanics are available.
   - Fornite. Psychological arousal increases as a result of the playable space shrinking across time. Players gradually get closer to one another, fighting for survival gets more intense, before hitting a crescendo.
   - Super Hexagon. Speed of shrinking, frequency of directional change, and complexity of puzzles slowly increases across time, thus increasing arousal until victory or failure.

**U4. AFFORDANCE:**

1. For most of the game, actions and actionable objects made available to the player are such that it is either immediately clear how they are to be used, or that the player will be able to figure out how said options are to be used. Discount puzzles-based objectives that are clearly indicative of requiring significantly more thought to solve.
   - Skyrim. Locked backdoors are frequently accompanied by nearby levers or switches, indicative that they are probably to be opened by interacting with said lever or switch.
   - Any game where compulsory objectives are based on a secret instead of a realistically solvable puzzle.

**U5. TRANSPARENCY:**

1. For most of the game, options made available to the player, in both game and user interface, requires little to no thought to discern their purpose. Discount from assessment mechanics and other processes that may be unique to the game; that require and have been given dedicated explanation.
   - In most games, selecting an audio button in a menu will reveal auto-related option available for adjustment.
   - Arcane shooter games with real world gun props. A reticule typically appears on screen to indicate where bullets will fire to.
   - Rebooted Tomb Raider games – climbable walls have white streaks on them to indicate their nature.

**U6. CONSISTENCY:**

1. How the player manages their interactions with the game remains mostly constant throughout. Includes predictable outcomes when controlling the game through external instruments (i.e. controllers, keyboards).
   - Most games have predictable outcomes when keys are pressed (e.g. press this button to talk to an NPC, press that button to pick up objects.)
   - Example of poor consistency – Fallout 4. From the beginning to the mid-late stage, pressing G for any amount of time will throw a grenade. After this stage, it becomes possible to select a skill that changes the button. Pressing G and holding it even slightly will now expend a valuable resource that causes a powerful enough explosion to kill the player. Change is sudden, abrupt, and extremely easy to accidentally cause.

**SERIOUS GAME MARKERS SCORING PROTOCOL**

**LEARNING**

**L1. ASSESSMENT:**

1. The game features a grading system to assess the performance of the player. Discount ‘total score’ features that do not make clear scoring systems.
   - Clinical setting simulation games. Player sees patients with humorous but real problems. End of the month ‘follow-up’ reports by nurses indicate some measure of ‘success rate’ of player’s chosen diagnoses/treatments.
   - Overwatch. Medals (bronze, silver, gold) are awarded for specific categories, such as damage dealt and healing done, at the end of the game as an indicator of performance relative to other players.

**L2. EXPECTATION DEFYING:**

1. Game attempts to prevent players from being conditioned to one stimulus to the point introducing a second, necessary stimulus has no effect. Operationally, the game avoids monotonous and predictable ‘learning moments’, and takes steps to keep players from knowing what will happen next with regards to learning. Also refers to attempts by the game to prevent the development of, or to disrupt blocking – where players become conditioned to a particular stimulus, such that another stimulus necessary for learning will not have the desired effect.
   - Medical simulation game. Player logically expects output of ‘Y’ from drug ‘X’ due to similarity with previous medications. Actual output is ‘Z’, accompanied by an explanation.
   - Blocking refers to a second stimulus being ‘blocked’ from association with an outcome due to a first stimulus already having been conditioned to that outcome. Novelty and expectation breaking can prevent this.

**L3. LOAD MANAGEMENT:**

1. Game scaffolds the delivery of novel information in an attempt to actively keep players within the zone of proximal development, and takes steps to avoid overloading the player.
   - Game aimed at teaching cellular function should gradually introduce the purpose of basic organelles *before* demonstrating impact on the human body.
   - Ideally, game should ensure mastery of a sub-topic before moving onto another topic that requires prior understanding.

**L4. RELEVANCE:**

1. The knowledge or skills the game intends to impart is presented in a manner and standard suitable for the target audience’s learning level and interests.
   - Medical emergency game targeted at computer-savvy medical students with damaged bodily structures/organs highlighted when injured patients enter a simulated emergency room. Computer-savvy medical students would know highlights are meant to draw attention to objects of interest, and thus focus on them.
   - The learning will not be relevant to the player if the critical knowledge (usually the one they will be assessed on) to be learnt, while present in the game, is not presented in a manner that can be understood by the player (i.e. complex puzzle games containing hidden-but-critical information being played by people who do not have time to solve puzzles).
   - If the knowledge is instead sequestered away in a manner that makes it very difficult or unrealistic for the player to dig out, then Relevance is scored as 0.

**L5. KNOWLEDGE:**

1. The game contains the knowledge and skills it intends for the player to intake in and utilise. Discount instructions on how the game is meant to be played.
   - Game whose intent is to ensure medical students correctly remember the diagnostic criteria for specific diseases must accurately feature said diagnostic criteria. It need not be a copy-paste of the ICD, but all the information must be present in the game.

**L6. TARGET OUTCOMES:**

1. The game has clear goals or target outcomes that it intends for the player to achieve. Discount considerations as to whether or not the game’s journey to this outcome is done well.
   - Healthy lifestyle games often operationalise the target outcome on a spectrum of ‘awareness’ – Latent awareness, constant awareness, behavioural modification attempts, successful behavioural modification
